# Mr. Edward Steinkopff

**Published:** 1906-05

**Authors:** 


MR. EDWARD STEINKOPFF, the founder of the Apolinaris business, died recently at his home at Lyndhurst, Hayward's Heath, England, in the sixty-ninth year of his age. He was born in Mecklenburg, married in Frankfort, and joined a German house in Glasgow in the early 70's. He suffered a reverse on the failure of the City of Glasgow bank, and then, in conjunction with the late George Smith, of Smith, Elder & Co., founded the Apolinaris business. In 1897 the business was sold to the late Frederick Gordon for nearly £2,000,000. Mr. Steinkopff took his own share of the purchase money, about a million sterling, in cash. He bought the charming estate of Lyndhurst, and spent the rest of his life in retirement there, with occasional brief periods of residence at his house in Berkeley Square, which at one time was the abode of the Premier Pitt.

